# Deciphering MET‐dependent modulation of global cellular responses to DNA damage by quantitative phosphoproteomics

**DOI:** 10.1002/1878-0261.12696

**Published:** 2020-05-13

**Authors:** Ariel Bensimon, Jonas P. Koch, Paola Francica, Selina M. Roth, Rahel Riedo, Astrid A. Glück, Eleonora Orlando, Andree Blaukat, Daniel M. Aebersold, Yitzhak Zimmer, Ruedi Aebersold, Michaela Medová

**Affiliations:** ^1^ Department of Biology Institute of Molecular Systems Biology ETH Zürich Switzerland; ^2^ Department of Radiation Oncology, Inselspital Bern University Hospital University of Bern Switzerland; ^3^ Department for BioMedical Research, Inselspital Bern University Hospital University of Bern Switzerland; ^4^ Global Research & Development Merck KGaA Darmstadt Germany; ^5^ Faculty of Science University of Zürich Switzerland; ^6^ Present address: CeMM Research Center for Molecular Medicine of the Austrian Academy of Sciences Vienna Austria

**Keywords:** ATM, DNA damage response, ionizing radiation, mass spectrometry, MET, receptor tyrosine kinase

## Abstract

Increasing evidence suggests that interference with growth factor receptor tyrosine kinase (RTK) signaling can affect DNA damage response (DDR) networks, with a consequent impact on cellular responses to DNA‐damaging agents widely used in cancer treatment. In that respect, the MET RTK is deregulated in abundance and/or activity in a variety of human tumors. Using two proteomic techniques, we explored how disrupting MET signaling modulates global cellular phosphorylation response to ionizing radiation (IR). Following an immunoaffinity‐based phosphoproteomic discovery survey, we selected candidate phosphorylation sites for extensive characterization by targeted proteomics focusing on phosphorylation sites in both signaling networks. Several substrates of the DDR were confirmed to be modulated by sequential MET inhibition and IR, or MET inhibition alone. Upon combined treatment, for two substrates, NUMA1 S395 and CHEK1 S345, the gain and loss of phosphorylation, respectively, were recapitulated using *invivo* tumor models by immunohistochemistry, with possible utility in future translational research. Overall, we have corroborated phosphorylation sites at the intersection between MET and the DDR signaling networks, and suggest that these represent a class of proteins at the interface between oncogene‐driven proliferation and genomic stability.

## Graphical abstract

Interference with the MET receptor tyrosine kinase signaling affects the cellular DNA damage response (DDR) to ionizing radiation. Using two proteomic techniques to explore phosphorylation signaling networks, we identified several substrates downstream of MET and the DDR that were modulated by sequential MET inhibition and IR, or MET inhibition alone.

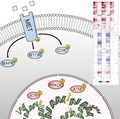

**Abbreviations**
ATM: ataxia telangiectasia mutated
ATR: ataxia telangiectasia and Rad3‐related protein
CDK: cell cycle‐dependent kinase
CHEK1: checkpoint kinase 1
CHEK2: checkpoint kinase 2
DDA: DNA‐damaging agent
DDR: DNA damage response
DNA‐PKcs: catalytic subunit of the DNA‐dependent protein kinase
ERK: extracellular signal‐regulated kinase
GO: gene ontology
IHC: immunohistochemistry
IR: ionizing radiation
KSRs: kinase–substrate relationships
MAPK: mitogen‐activated protein kinase
METi: MET inhibitor EMD1214063
MS: mass spectrometry
mTOR: mammalian target of rapamycin
PI3K: phosphoinositide 3‐kinase
PIKKs: phosphatidylinositol 3‐kinase‐related kinases
RSKs: ribosomal protein S6 kinases
RTK: receptor tyrosine kinase
SRM: selected reaction monitoring
WB: western blotting

## 1 Introduction

The DNA damage response (DDR) involves intricate signaling networks of checkpoint and repair pathways that respond to various forms of genotoxic stress to restore and sustain genome integrity or promote cellular death. The cellular response to genotoxic stress is largely governed by three members of the family of phosphatidylinositol 3‐kinase‐related kinases (PIKKs), ataxia telangiectasia mutated (ATM), ataxia telangiectasia and Rad3‐related protein (ATR), and the catalytic subunit of DNA‐dependent protein kinase (DNA‐PKcs or PRKDC) (Dery and Masson, 2007; Goldstein and Kastan, 2015; Polo and Jackson, 2011). In addition to the PIKKs and two other prominent DDR kinases, checkpoint kinase 1 (CHEK1) and checkpoint kinase 2 (CHEK2), numerous other kinases have been implicated in the response to genotoxic stress (Bensimon *et al*., 2011; Smith *et al*., 2010). Aiming to promote cellular death by DDR activation, DNA‐damaging agents (DDAs), such as chemotherapy or ionizing radiation (IR), have been long used in cancer treatment (Goldstein and Kastan, 2015). Furthermore, numerous agents sensitizing cancer cells to DDAs are currently under both preclinical and clinical investigations and hold great promise as anticancer modalities, alone, or as combination therapies (Al‐Ejeh *et al*., 2010; Lord and Ashworth, 2012; O'Connor, 2015).

Different oncogenes activated during cancer development can lead to DDR activation as a consequence of excess replicative stress, which may result in genomic instability and alterations in checkpoint and repair mechanisms (Hills and Diffley, 2014; Tian *et al*., 2015). For example, deregulated growth factor receptor tyrosine kinase (RTK) signaling is a prominent hallmark of numerous cancers and is firmly associated with persistent growth and proliferation signals which promote tumor onset, progression, and metastasis (Hanahan and Weinberg, 2011; Lemmon and Schlessinger, 2010). Accordingly, remarkable advances have been made over the last two decades in the discovery and clinical development of a wide range of molecular entities targeting RTKs (Tibes *et al*., 2005), some of which were found to enhance sensitivity to DDAs (e.g., Meyn *et al*., 2009). One such RTK possibly involved in the DDR is MET, the RTK for hepatocyte growth factor (HGF) which is deregulated in abundance and/or activity in a variety of human tumor types (Matsumoto *et al*., 2017) and serves as an oncogenic target (Peters and Adjei, 2012). Like other RTKs, MET activates multiple downstream phosphorylation signaling pathways including the phosphoinositide 3‐kinase (PI3K)/AKT/mammalian target of rapamycin (mTOR) and the mitogen‐activated protein kinase (MAPK)/extracellular signal‐regulated kinase (ERK) cascades (Garajova *et al*., 2015). Over the years, reports have indicated links between MET and the DDR: MET inhibition enhanced sensitivity to IR (De Bacco *et al*., 2016; Medova *et al*., 2010; Welsh *et al*., 2009), at least in part due to impaired DNA repair (Medova *et al*., 2012), and in the clinic, aberrant MET expression and activity have been associated with patient treatment outcome following radiation therapy (Baschnagel *et al*., 2014; Bhardwaj *et al*., 2013). We reason that better understanding of how MET signaling modulates the DDR can translate in the future to a better understanding of combination therapies in the clinic.

Phosphorylation signaling networks modulated by MET inhibition (Bertotti *et al*., 2009; Moritz *et al*., 2010) or by DNA damage (Beli *et al*., 2012; Bennetzen *et al*., 2010; Bensimon *et al*., 2010; Matsuoka *et al*., 2007) have been explored using various proteomic techniques, including mass spectrometry (MS)‐based phosphoproteomics. In this study, we aimed to identify proteins at the intersection between these networks, by deciphering how MET inhibition modulates the cellular phosphorylation response to IR. To conduct an initial discovery survey, we focused on potential PIKK substrates, using a motif‐directed immunoaffinity‐based MS approach (Stokes *et al*., 2012). From our survey, candidate phosphorylation sites on proteins involved in DNA damage, cell cycle, and cellular metabolism were selected for characterization by targeted MS. Selected reaction monitoring (SRM) assays were developed for these candidate sites along with numerous known substrates involved in these signaling networks, and quantitative proteomic analyses were performed in multiple perturbations in several cell lines. Last, selected phosphorylation sites at the intersection between these networks were further investigated, including NUMA1 and CHEK1, phosphorylated at S395 and S345, respectively.

## 2 Materials and methods

### 2.1 Cell lines

Human gastric carcinoma cell line GTL‐16 was provided by P. Comoglio (Medical School University of Torino, Italy), nonsmall cell lung cancer cell line EBC‐1 by S. Giordano (University of Torino, Torino, Italy), and pharyngeal carcinoma line Detroit 562 was purchased from American Type Culture Collection (ATCC, Manassas, VA, USA). GTL‐16 and EBC‐1 cells were cultured in RPMI (Gibco, Invitrogen, Reinach, Switzerland) supplemented with 5% and 10% FCS (Sigma‐Aldrich Inc., St. Louis, Missouri, USA), respectively, and an antibiotic/antimycotic solution (penicillin 100U·mL^−1^, streptomycin sulfate 100U·mL^−1^, amphotericin B as fungizone 0.25µg·mL^−1^; Gibco). Detroit 562 cells were maintained in MEM (Sigma‐Aldrich) supplemented with 10% FCS, nonessential amino acid solution (1% vol./vol.; Sigma‐Aldrich), and antibiotic/antimycotic solution.

### 2.2 Inhibitors

The MET small molecule inhibitor tepotinib (EMD1214063, MSC‐2156119; Merck Serono, Darmstadt, Germany (Bladt *et al*., 2013)) was added to cells at a concentration of 50 nm in all *in vitro* studies unless otherwise specified. KU55933 (ATM inhibitor), VE‐821 (ATR inhibitor), KU57788 (PRKDC inhibitor), AZD6244 (ERK inhibitor), and AZD5363 (AKT inhibitor; all from Selleck, Houston, TX, USA) were used at a final concentration (f.c.) of 10 µm. GDC0941 [PI3K inhibitor (Selleck, Houston, TX, USA)] was used at f.c. of 1 µm. Inhibitors were dissolved in DMSO, and working solutions were prepared freshly and remained in the media for the duration of the respective experiment.

### 2.3 Antibodies

MET Tyr1234&1235 (#3077), NUMA1 Ser395 (#3429), phospho‐Ser/Thr‐Gln‐Gly (#6966), phospho‐Ser‐Gln (#9607), phospho‐Thr(Asp/Glu)X(Asp/Glu) (#BL4176), phospho‐Thr‐X‐Arg (#2351), CHEK1 Ser345 (#2341), CHEK1 (2G1D5; #2360S), p90RSK Ser380 (#9335), PathScan® (Cell Signaling Technology) Multiplex Western Cocktail I [phospho‐p90RSK, phospho‐Akt, phospho‐p44/42 MAPK (Erk1/2), phospho‐S6; #5301], cleaved caspase‐8 (Asp391; #9496), cleaved caspase‐3 (Asp175; #9661), 53BP1 (#4937), ACIN1 (#4934), cdc2 (#9116T), cell cycle‐dependent kinase 2 (CDK2; #2546T), H2AX (#7631S), 11 (#8967), SMC3 (#5696), and MET (#8198S and #4560) antibodies were all obtained from Cell Signaling Technology (Danvers, MA, USA). Histone H2AX Ser139 (#05‐636), ATM Ser1981 (#05‐740), histone H3 Ser10 (#06‐570), and β‐actin (#MAB1501) antibodies were obtained from Merck Millipore Corporation (Darmstadt, Germany). TIF1B (KAP1) Ser824 (#A300‐767A) antibody was purchased from Bethyl Laboratories, Inc. (Montgomery, TX, USA). SMC3 Ser1083 (#NB100‐653) and ATM (#NB100‐309) antibodies were obtained from Novus Biologicals (Littleton, CO, USA).

### 2.4 Irradiation

In all *in vitro* studies, cells were irradiated (single dose of 10 Gy) using a ^137^Cs source (Gammacell 40; MDS Nordion, Ottawa, ON, Canada) at a dose rate of 0.86 Gy·min^−1^.

### 2.5 Cell proliferation assay

Cells were seeded in 24‐well plates (GTL‐16: 30000cells per well, EBC‐1: 40000cells per well, Detroit 562: 15000cells per well) and were treated with EMD1214063 (24h after seeding) and irradiated (48h after seeding) as indicated. Control samples were treated with DMSO at the corresponding time points. Seventy‐two hours after irradiation, the cells were fixed and stained with 2% crystal violet dissolved in acetic acid/methanol (2:1) for 30min at room temperature. The dye was removed, and the plates were washed with water. The plates were scanned, and cell density was calculated using imagej (http://imagej.nih.gov/ij/). The area of treated cells was normalized to the area of nontreated controls. Experiments were performed three times. Statistical significance was calculated with the graphpad prism Software (GraphPad, San Diego CA, USA) using a two‐way ANOVA test (*P<0.05; **P<0.01; ***P<0.001).

### 2.6 Western blotting

Cells were lysed in a buffer containing 20mm HEPES (pH 8.0), 9.0m urea, 1mm sodium orthovanadate, 2.5mm sodium pyrophosphate, and 1mm β‐glycerol‐phosphate. Lysates were sonicated and cell extracts cleared by centrifugation. Total protein concentration was determined using the Bio‐Rad protein quantification reagent (Bio‐Rad Laboratories, Inc., Hercules, CA, USA). Total protein extracts (20–100µg) were resolved by SDS/PAGE, transferred onto PVDF membranes (Novex™; Thermo Fisher Scientific), and blotted by standard procedures. Western blots for GTL‐16 and EBC‐1 cells using phospho‐Ser/Thr‐Gln‐Gly and Phospho‐Ser‐Gln motif antibodies along with β‐actin and MET Tyr1234&1235 were performed by Cell Signaling Technology (KinomeView Service).

### 2.7 Immunohistochemistry

Tissue samples were cut into 5‐μm sections using Leica Biosystems CM 3050S Research Cryostat (Leica Biosystems AG, Muttenz, Switzerland), and immunohistochemistry (IHC) was performed as described (Francica *et al*., 2016). Briefly, sections were fixed in 4% paraformaldehyde (PFA) and extracted in 1× PBS/Triton‐X 0.1%. Indicated primary rabbit antibodies were incubated at room temperature (RT) in blocking solution (1% normal goat serum in PBS‐T) overnight, followed by secondary antibody at RT for 1 h. Signal was detected using the Vectastain ABC Kit (Vector Laboratories Inc., Burlingame, CA, USA) and 3,3′‐diaminobenzidine (DAB; Sigma‐Aldrich), according to the manufacturer's instructions, followed by counterstaining with hematoxylin (Sigma‐Aldrich) for 1 min. Dehydrated sections were mounted using Eukitt (Kindler, GmbH, Freiburg, Germany). Images were obtained with a Leica DMRB microscope (Leica Microsystems, Wetzlar, Germany) at both 20× and 40× magnifications and manually quantified using the imagej software (http://imagej.nih.gov/ij/). Statistical analysis was performed with the GraphPad Prism Software using one‐way ANOVA test (*P < 0.05; **P < 0.01; ***P < 0.001).

### 2.8 *In vivo* model

EBC‐1 xenografts (10^6^ cells injected subcutaneously on the right flank) were grown in 12‐week‐old female Rag2 common gamma‐null mice (Rag2^−/−^γc^−/−^, Taconic), and the growth of the tumors was regularly monitored by caliper measurements. Once tumors had reached a size of 300 mm^3^ (day 0), mice were randomly divided into four treatment groups: vehicle control (Solutol HS 15; BASF ChemTrade GmbH, Burgbernheim, Germany), tepotinib only (15 mg·kg^−1^·day^−1^ *per os*, days 1–6), IR only (one single 6 Gy dose on day 3), and combination of tepotinib with IR. Radiation was delivered locally using the XStrahl 150 (Xstrahl Limited, Surrey, UK). Tumors were harvested 24 h after the last tepotinib treatment, frozen in Tissue‐Tek optimum cutting temperature medium (Sakura Finetek Europe B.V., Alphen aan den Rijn, The Netherlands), and stored at −20 °C. Animal experiments were conducted in strict compliance with Swiss Federal guidelines and have been approved by Federal Food Safety and Veterinary Office.

### 2.9 Discovery phosphoproteomics

#### 2.9.1 Peptide immunoaffinity enrichment

**Fig. 1:**
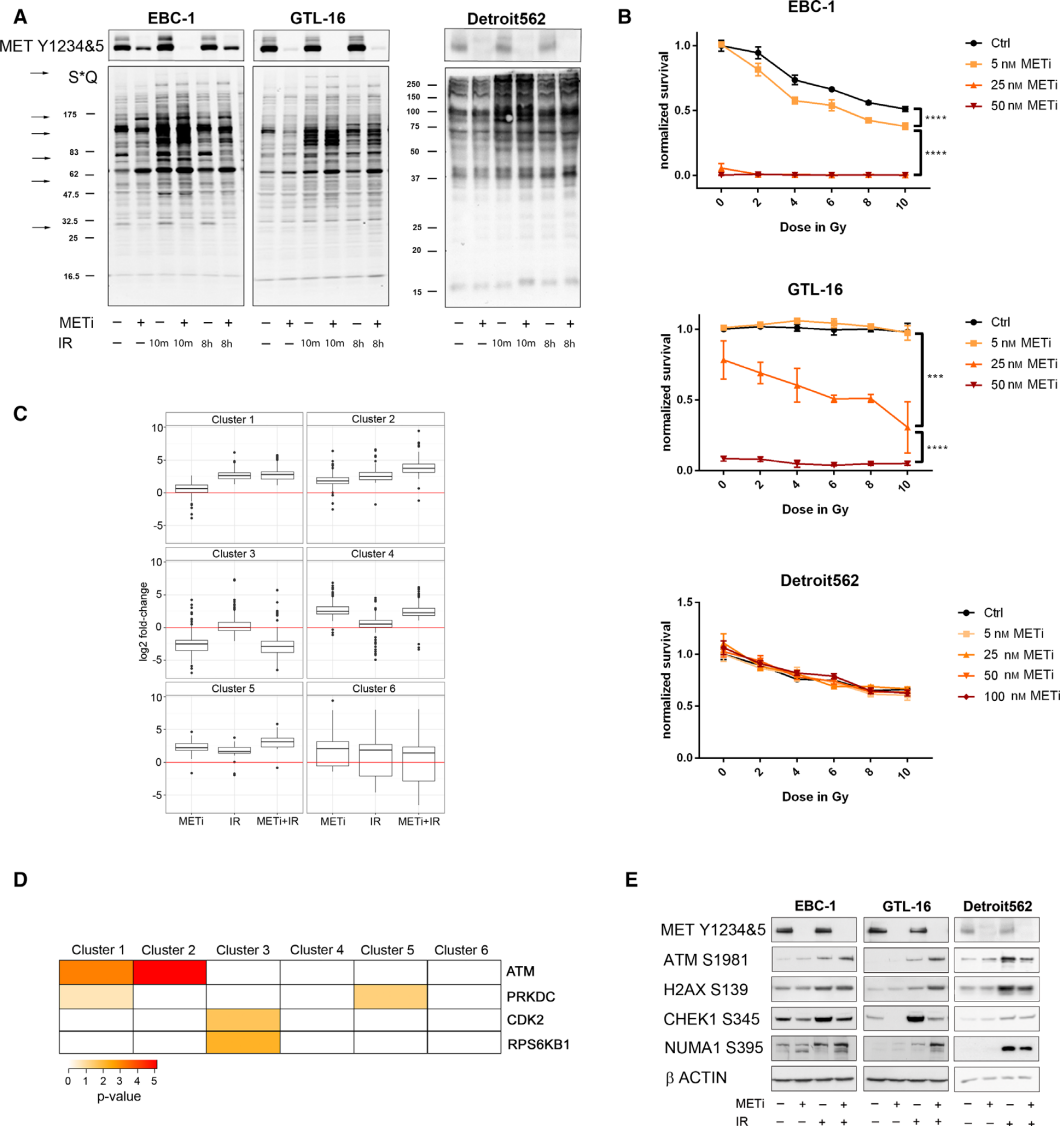
Immunoaffinity‐based phosphoproteomics identified phosphoproteins modulated by METi and IR. (A) Modulation of S*Q phosphorylation motif‐containing substrates (* denotes S phosphorylation) upon METi (EMD1214063, at a final concentration of 50nm, 16h prior to IR), IR (at a single dose of 10Gy), or their combination at 10min or 8h post‐IR. Arrows point at examples of prominent phosphorylation changes. MET autophosphorylation was blotted separately as a control for MET inhibition. (B) Survival curves for EBC‐1, GTL‐16, and Detroit 562, upon single IR doses alone and in combination with METi, based on the crystal violet staining assay (*n*=3; ±SD). Statistical significance was calculated with the graphpad prism Software using a two‐way ANOVA test (*P<0.05; **P<0.01; ***P<0.001; ****P<0.0001). (C) Five hundred fifty‐three phosphopeptides, identified in the PTMScan dataset as regulated by METi‐ and/or IR, were clustered based on their quantitative changes across conditions (*n*=1; Log_2_FC). (D) Association of the significant predicted putative kinases with the identified clusters. (E) Selected DDR phosphorylation sites are differentially impacted by METi in MET‐overexpressing cancer cell lines. Cells were treated with METi (for 24h), IR, or their combination (METi added 16h prior IR), lysed 8h post‐IR, and analyzed using antibodies against selected phosphorylation sites based on our dataset.

Two motif‐directed immunoaffinity enrichments were performed sequentially as described in Stokes *et al*. (2012) by Cell Signaling Technology (PTMScan Discovery Proteomics Services) from EBC‐1 cells. In the first immunoaffinity enrichment experiment, denoted as ‘Atypical’, a preprepared mix of these motif‐directed antibodies was used: phospho‐Ser‐Gln (#9607; see Fig.1A for a representative western blot), phospho‐Ser/Thr‐Gln‐Gly (#6966; Fig.S1A), phospho‐Thr‐(Asp/Glu)‐X‐(Asp/Glu) (#5808; Fig.S1B), and phospho‐Thr‐X‐Arg (#2351; Fig.S1C). Upon request, a second immunoaffinity enrichment experiment, denoted as ‘PIKK’, was performed with these two antibodies: phosphor Ser‐Gln (#9607; Fig.1A) and phospho‐Ser/Thr‐Gln‐Gly (#6966; Fig.S1A).

#### 2.9.2 Data acquisition and processing

LC‐MS/MS analysis was performed as described in Stokes *et al*. (2012), with the following parameters: A 72‐min linear gradient was utilized. Acquisition on an LTQ Orbitrap Velos (Thermo Fisher, San Jose, CA, USA) was performed using the following MS parameter settings: MS run time 96 min, MS1 scan range (300.0–1500.00), and top 20 MS/MS (min signal 500, isolation width 2.0, normalized coll. energy 35.0, activation‐Q 0.250, activation time 20.0, lock mass 371.101237, charge state rejection enabled, charge state 1+ rejected, dynamic exclusion enabled, repeat count 1, repeat duration 35.0, exclusion list size 500, exclusion duration 40.0, exclusion mass width relative to mass, exclusion mass width 10 p.p.m.).

In each immunoaffinity experiment, the four samples were acquired twice as technical replicates. The MS data files were provided by Cell Signaling Technology. The files were processed for identification and quantification using maxquant and andromeda version 1.5.2.8 (Cox and Mann, 2008; Cox *et al*., 2011), with the following settings: variable modifications: oxidation (Met) and phosphorylation (Ser/Thr/Tyr); peptide FDR set to 0.01; site localization FDR set to 0.1; and ‘Match between runs’ enabled, with a time window of 1 min; the search was performed against the human UniProt FASTA dataset UP000005640 (both canonical and additional sequences). The two ‘modification‐specific’ output tables (Table S1) were processed using perseus version 1.5.2.6 (Tyanova *et al*., 2016). In brief, the data were normalized and missing values were imputed from a normal distribution. Only phosphopeptides that matched at least one of the above‐mentioned antibody motives (based on site localization) were considered for further analysis. Finally, the tables of the two immunoaffinity enrichments were merged (Table S2). We termed as ‘regulated’ those phosphopeptides which are regulated by a fold change > 3 in at least two out of three conditions. Of note, since the two enrichments were performed from a single biological replicate, we did not test the statistical significance of these fold changes.

#### 2.9.3 Data analysis

Unless otherwise stated, calculations were performed in r version 3.2.3 (R Core Team, 2015) and plotted using ggplot2 (Wickham, 2009). Gene ontology (GO) analysis was performed using the r package clusterprofiler 3.05 (Yu *et al*., 2012) and a hypergeometric test was used as an enrichment test, with all proteins in Table S1 as the background proteome. Phosphopeptides that manifest similar patterns of regulation were clustered using the algorithm CLICK, which is available in the Expander tool version 7 (Sharan *et al*., 2003) with an expected mean homogeneity of at least 0.95. Putative kinases were predicted using the online tool networKIN (Horn *et al*., 2014), and for each phosphorylation site, the highest scoring predicted kinase was retained. Enrichment of predicted kinases was assessed using the hypergeometric test followed by adjustment for multiple testing using the Benjamini–Hochberg method (Benjamini and Hochberg, 1995). In addition to predicted kinase–substrate relationships (KSRs), known KSRs were retrieved from phosphositeplus (Hornbeck *et al*., 2015), KEGG (Kanehisa *et al*., 2017), and iptmnet (Ross *et al*., 2017) and uniprot (UniProt, 2015). Networks were illustrated using cytoscape version 3.3.

### 2.10 Targeted phosphoproteomics

To enable targeted quantification of selected phosphopeptides across a larger set of samples, we chose to perform phosphoproteome enrichment by titanium dioxide rather than immunoaffinity‐based enrichment (von Stechow *et al*., 2015).

#### 2.10.1 Phosphopeptide enrichment by titanium dioxide

Frozen cell pellets were resuspended in 8m urea solution containing 0.1m ammonium bicarbonate (ABC), protease inhibitor cocktail (Roche, Basel, Switzerland), and phosphatase inhibitor cocktails 2 and 3 (Sigma‐Aldrich), and the resulting extracts were spun for 10min at 37°C, 150 ***g***, Bicinchoninic acid protein assay (Thermo Scientific, Rockford IL, USA) was used for determining protein concentrations. Lysates were reduced with tris(2‐carboxyethyl)phosphine (Sigma‐Aldrich), alkylated with iodoacetamide (Sigma‐Aldrich), and digested over night at 37°C with sequencing‐grade modified trypsin (Promega, Madison WI, USA) at a protein‐to‐enzyme ratio of 50:1. Peptides were desalted on SEP‐PAK C18 cartridge (Waters, Milford MA, USA) and dried under vacuum. Phosphopeptides were isolated from 1 to 1.8mg of total peptide mass using TiO_2_ by a protocol modified from Bodenmiller *et al*. (2007) and Zhou *et al*. (2013). Briefly, the dried peptides were dissolved in an 80% acetonitrile (ACN), 2.5 % TFA solution saturated with phthalic acid (Sigma‐Aldrich), and then incubated with TiO_2_ for 30min with end‐over‐end rotation. The beads were transferred to a 200‐μL tip and processed as described in Zhou *et al*. (2013). Eluted phosphopeptides were desalted using C18 ultramicrospin columns (Nest, Southborough, MA, USA) and resuspended in a 2% ACN/0.1% FA buffer that contained diluted synthetic reference peptide mix (see below) and iRT retention time kit (Escher *et al*., 2012).

#### 2.10.2 Targeted data acquisition

Peptide samples were analyzed on a 5500QTRAP hybrid triple quadrupole/ion trap mass spectrometer (SCIEX, Concord, Canada) equipped with a nanoelectrospray ion source. Chromatographic separation was performed by a NanoLC AS2 (SCIEX) coupled to a 15‐cm (75 μm ID) fused silica emitter (MSwil, Zurich, Switzerland), self‐packed with ProntoSIL C18 AQ 3 μm resin (WICOM International GmbH, Maienfeld, Switzerland). Peptides were separated at a flow rate of 300 nL·min^−1^, in a gradient of solvent A (98% water/2% ACN/0.1% FA) and solvent B (98% ACN/2% water/0.1% FA; a 55 min gradient, 2–40%, or a 35 min gradient, 2–35%). The instrument was operated in scheduled positive SRM mode at a unit resolution (0.7 *m*/*z* half‐maximum peak width) for both Q1 and Q3 analyzers. Unless further optimized, collision energies (CEs) were calculated according to the formulas: CE = 0.044 × *m*/*z* precursor + 5.5 and CE = 0.051 × *m*/*z* precursor + 0.55, for doubly and triply charged precursor ions, respectively.

#### 2.10.3 Targeted assay generation

Synthetic phosphopeptides (Thermo Scientific) labeled with heavy isotopes at the C‐terminal Lys (+8 Da) or Arg (+10 Da; unless otherwise mentioned in Table S2) were pooled (~ 15–25 peptides per pool) and used for assay generation. The successful synthesis of each peptide was first confirmed in shotgun proteomic analysis. Subsequently, the pools were analyzed on a 5500QTRAP to generate full MS2 fragment ion chromatograms. The fragment ions of the full 1+ and 2+ y‐ion and b‐ion series of the 2+ and 3+ precursors were acquired with CEs calculated as described above. Thus, on average, several dozens of transitions were measured per synthetic peptide. For selected phosphopeptides, neutral loss (−98 Da) ions were also included in the transition list. Skyline (MacLean *et al*., 2010) and Panorama (Sharma *et al*., 2014) were used to generate a library from the acquired full MS2 fragment ion chromatograms. For each synthetic phosphopeptide, we selected the 4–8 most intense fragment ions, taking into account phosphorylation site localization.

#### 2.10.4 Targeted data analysis

Skyline (MacLean *et al*., 2010) was used for targeted data analysis (version 3.5). SRM peak integration was manually confirmed, and interfered transitions were removed. The reference synthetic standards were used to validate peptide identity by analogy of chromatographic and fragmentation properties to the reference (rdotp > 0.9). Relative quantification and statistical analysis were performed using msstats (version 3.3.4) (Choi *et al*., 2014) with the following modifications to default parameters: Normalization was performed by equalizing the medians of the reference synthetic standards; Tukey’s median polish was used as summary method. An adjusted *P*‐value of 0.05 was set as a cutoff for significance. Of note, in the second SRM experiment, singly phosphorylated H2AX was quantified with rdotp < 0.9 due to chromatographic issues with two of the synthetic standards for H2AX.

### 2.11 Phosphoproteomic data deposition

The MS proteomic data have been deposited to the ProteomeXchange Consortium via the PRIDE (Vizcaino *et al*., 2016) partner repository with the dataset identifier http://www.ebi.ac.uk/pride/archive/projects/PXD009433.

## 3 Results

### 3.1 MET inhibition modulates phosphorylation levels on putative PIKK substrates

We have reported (Medova *et al*., 2010) that in MET‐overexpressing human gastric adenocarcinoma cells GTL‐16, inhibiting MET with the small molecule PHA665752 16 h prior to IR treatment led to a substantial increase in the phosphorylation levels of both H2AX S139, a prominent substrate of ATM, ATR, and PRKDC (Friesner *et al*., 2005; Stiff *et al*., 2004), and ATM S1981, a marker of the activated form of this kinase (Shiloh and Ziv, 2013). These increases suggested that phosphorylation events may lay at the intersection between MET and DDR signaling, and possibly represent a broader phenomenon in cellular DDR. Initially, we evaluated the impact of MET inhibition on global changes in phosphorylation by western blotting (WB), using antibodies recognizing two known sequence motifs associated with PIKKs: phospho‐Ser‐Gln (S*Q) and phospho‐Ser/Thr‐Gln‐Gly ((S/T)*‐QG). Two wild‐type MET‐overexpressing human cancer cell lines, gastric adenocarcinoma cell line GTL‐16, and the nonsmall cell lung cancer line EBC‐1 (Smolen *et al*., 2006) were first treated for 16 h with the specific MET inhibitor EMD1214063 (termed METi hereforth), IR (at a single dose of 10 Gy), or the combination, and were harvested at two time points (10 min and 8 h). In the resulting blots, we found multiple bands which increase/decrease upon METi treatment alone or in combination with IR (Fig. 1A and Fig. S1A). We did not observe such changes upon METi treatment in the pharyngeal carcinoma cell line Detroit 562 (Fig. 1A, Fig. S1A), another MET‐overexpressing cell line displaying HGF‐independent basal MET Y1234&5 phosphorylation, suggesting that METi‐dependent changes occur only in particular MET‐expressing cell lines and correlate well with their sensitivity toward METi and with METi‐induced radiosensitization (Fig. 1B).

To identify and quantify the phosphorylation sites modulated by METi in the context of DNA damage, a discovery phosphoproteomic screen was carried out by motif‐directed immunoaffinity enrichment followed by LC‐MS/MS analysis (PTMScan Direct) (Stokes *et al*., 2012). EBC‐1 cells were treated with METi alone (24 h), IR alone (8 h), or the sequential combination of both (METi added 16 h prior IR). From these four samples, two independent phosphopeptide immunoaffinity enrichments were performed using two different premixed immunoaffinity resins against the motives: S*Q, (S/T)*‐QG, T*‐D/E‐X‐D/E, and T*‐X‐R (representative WBs utilizing these antibodies are shown in Fig. 1A and Fig. S1A–C). Changes in phosphorylation levels across samples were assessed by label‐free quantification using MaxQuant (Cox and Mann, 2008). For our analysis, we considered only those phosphopeptides that matched at least one of the motives used for enrichment in the experiments, as estimated by the probability of correct site assignment. The final dataset included 1605 phosphopeptides, mapping to 757 proteins (Tables [Link], [Link]). One thousand one hundred ninety‐four phosphopeptides were phosphorylated on at least one S/T‐Q motif, while the remaining phosphopeptides were phosphorylated on one of the other motives described above. Among the 1605 identified phosphopeptides, 564 changed by at least threefold in their abundance in at least two of the three treated samples compared with the untreated sample and were selected as regulated phosphopeptides for further analysis. These phosphopeptides mapped to 318 proteins, which were enriched for GO terms (Fig. S1D) including ‘nucleic acid metabolic process’, ‘DNA repair’, or ‘regulation of mitotic cell cycle’, as would be expected given our experimental design.

Out of the 564 regulated phosphopeptides, 553 phosphopeptides subdivided into six clusters by the similarity of their quantitative changes across conditions (Fig. 1C and Table S2). From the average pattern of phosphorylation changes, it appears that phosphopeptides in cluster 1 were to a large extent upregulated by IR alone, while phosphopeptides in clusters 3 and 4 were down‐ or upregulated, respectively, by METi. Interestingly, clusters 2 and 5 pointed to two groups of phosphopeptides that were upregulated by METi alone and by IR alone. Phosphopeptides in cluster 2 were further upregulated by the combination of METi with IR as compared to IR alone. Several known IR‐induced phosphorylations were distributed between these clusters, particularly in clusters 1 and 2, for example, ATM S1981, NBN S343, and RAD50 S635. As expected from IR‐induced clusters, clusters 1 and 2 were enriched for GO terms such as ‘double‐strand break repair’ and ‘chromosome organization’. However, only cluster 1 was enriched for ‘DNA replication’, ‘DNA damage checkpoint’, and ‘transcription from RNA polymerase II promoter’, whereas clusters 3–6 were not enriched for any GO term (Fig. S1E).

Between all clusters, based on phosphositeplus (Hornbeck *et al*., 2015), a particular kinase has been documented to phosphorylate a site on only 84 out of the 564 regulated phosphopeptides. The most prominent kinases with assigned substrates in our dataset were ATM/ATR/PRKDC (44 phosphopeptides) and CDK1/2/4/6 (33 phosphopeptides). To complement known kinases, we used NetworKIN (Horn *et al*., 2014) to predict putative kinases for each of the phosphorylation sites and tested which predicted kinases are enriched in each of the clusters. Clusters 1 and 2 were significantly enriched for predicted ATM substrates (*P*‐values 7*10^−5^ and 7*10^−6^, respectively, Fig. 1D and Table S3), while PRKDC was enriched in cluster 5 (*P*‐value 0.0465), which contained a phosphopeptide corresponding to PRKDC itself. Last, modulated by METi alone, cluster 3 was enriched for CDK2 and RPS6KB1 (also known as p70S6K; *P*‐values 0.02 and 0.006, respectively). Of note, few substrates were predicted or known for these kinases in our dataset, but these included RB1, a substrate of CDKs, and RPS6, a substrate of ribosomal protein S6 kinases (RSKs). To visualize the relationships between the enriched kinases and the respectively mediated phosphorylation sites, we generated a network of substrates connected to the predicted kinases enriched in this dataset or to their known upstream kinases (Fig. S2). Twelve kinases were included in the network, associated with 115 phosphorylation sites. In addition to known ATM substrates, 69 phosphorylation sites were predicted as substrates of ATM in this network. As is apparent from the results, putative ATM‐predicted sites spanned all the clusters mentioned above. Hence, this network pointed to ATM activity as the most dominant in numbers and breadth in the context of METi‐modulated response to DNA damage. Yet, as numerous regulated phosphopeptides were not associated with any of these kinases, we hypothesized that the underlying signaling networks extend beyond the few kinases enriched in our dataset.

To assess the possibility that the enriched kinase activities are modulated by METi treatment as emerged from the cluster analysis, we repeated the experiment in all three wild‐type MET‐expressing cell lines named above and analyzed by WB phosphorylation sites selected based on the screen. In both EBC‐1 and GTL‐16 cells but not in Detroit 562, the levels of ATM S1981 and NUMA1 S395 were higher in cells treated with the combination as compared to IR alone (Fig. 1E), recapitulating the mean pattern observed for phosphopeptides in cluster 2. A similar pattern was also observed for H2AX S139 in GTL‐16 at least. Since no antibody was available for the CHEK1 phosphorylation site S365, we utilized an antibody against the phosphorylation site S345 on CHEK1. CHEK1 S345 was detectable in untreated EBC‐1 and GTL‐16 cells and reduced upon METi (whether alone or in combination with IR). Last, we found that in EBC‐1 cell line, downregulation of RPS6 S235&6 was indeed MET‐dependent and IR‐independent, and is likely the consequence of inhibiting several kinases that are upstream of RSKs (Fig. S3A).

These observations by WB led us to assume that the quantitative patterns that we observed in the discovery screen are of biological significance. Hence, MET inhibition may modulate DDR signaling by influencing the activities of kinases directly involved in DDR such as ATM, PRKDC, CHEK1, and possibly ATR. Of note, in accordance with the roles of MET in regulating proliferation, we found that combined treatment led to a rapid decrease in phosphorylated histone 3, a marker for proliferating cells (Fig. S3B) (Factor *et al*., 2010; Rebouissou *et al*., 2017). Thus, given that MET inhibition leads to cell cycle arrest in these cells (Medova *et al*., 2010; Zhang *et al*., 2014), DDR signaling may be modulated also indirectly, via kinase activities involved in cell cycle (e.g., CDKs) and cellular growth (e.g., RSKs).

### 3.2 Targeted phosphoproteomics validates METi modulation of IR‐induced phosphorylations

**Fig. 2:**
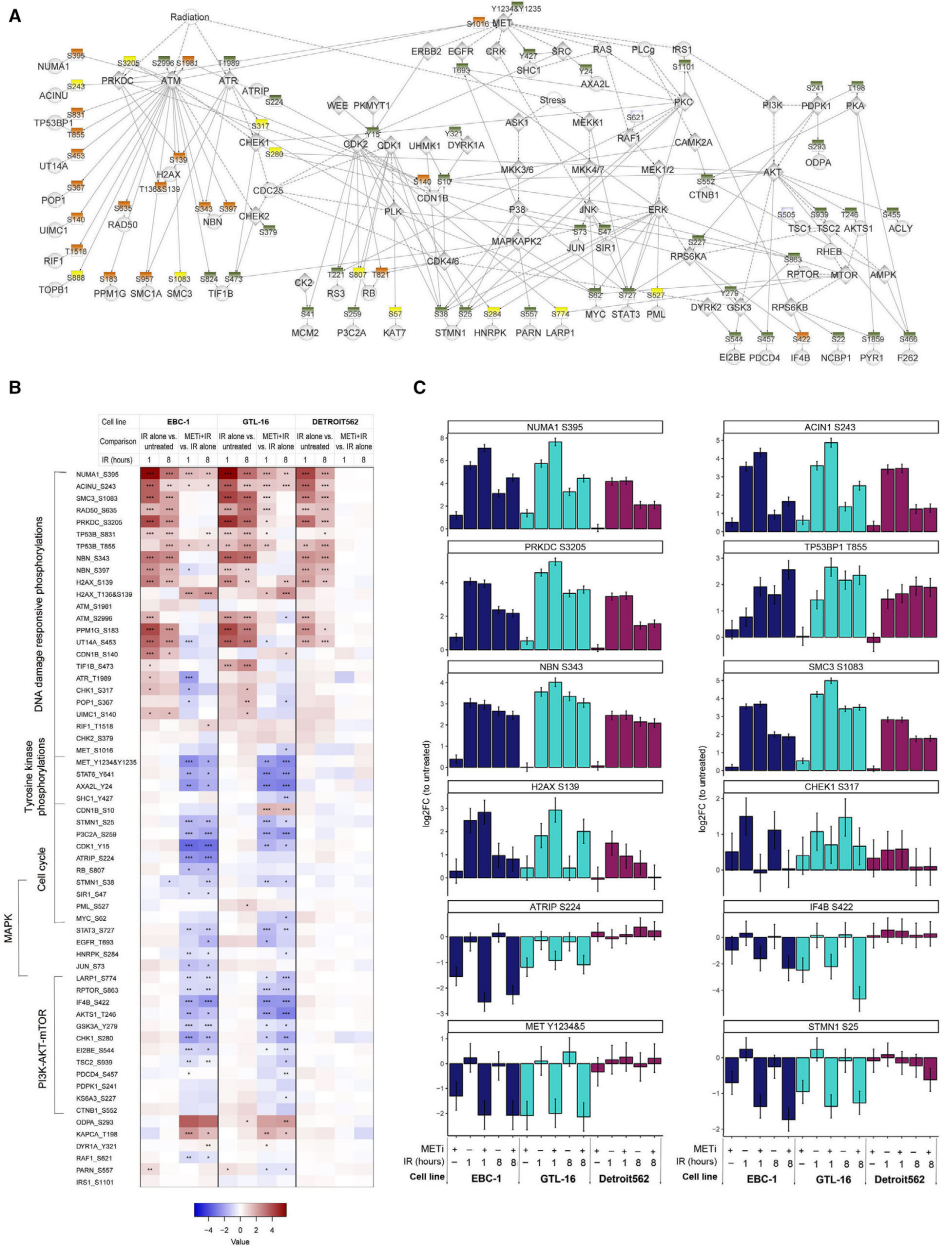
METi‐ and IR‐related phosphorylations validated by targeted proteomics. (A) Phosphorylation sites selected for analysis by targeted proteomics. Phosphorylation sites identified in discovery screen (orange squares), phosphorylation sites on proteins identified in the discovery screen (yellow squares), and phosphorylation sites selected from literature (green squares) are linked to their known (full edges) or predicted (dashed edges) kinases. (B) METi‐based modulation of cellular DDR monitored by SRM. Assays were monitored in samples treated with METi, IR, or combination of both. Here are presented the results from statistical comparisons addressing two questions: Which are the significant changes in response to IR alone, compared with control; which are the significant changes in response to the combination of METi and IR, compared with IR alone. Log_2_FC (*n* = 3) is represented by a color code; adjusted *P*‐values by symbol: <0.0005, ‘***’; <0.005, ‘**’; <0.05, ‘*’. (C) Log_2_FC with standard error bars per condition is presented for selected phosphorylations in comparison with control sample (untreated) within each cell line (*n* = 3). Note the significant difference between IR alone and the combination for NUMA1 S395, ACIN1 S243 in selected time points and cell lines, as opposed to NBN S343.

To further investigate the hypotheses generated from our dataset, without constraining ourselves to available antibodies, we decided to utilize targeted proteomics by SRM. In SRM, specific mass spectrometric assays are generated *a priori* for each targeted phosphopeptide (e.g., from a fragment ion spectrum of a synthetic phosphopeptide) and these assays are then used to selectively detect and quantify dozens of such peptides of interest in multiple biological samples (Picotti *et al*., 2010). For the development of SRM assays, we selected candidate phosphopeptides from the discovery screen, as well as phosphopeptides from literature, which were mapped to known substrates as surrogate markers for the activation/activity state of the candidate kinases. First, by targeting candidates selected from the screen, we aimed to confirm modulation of IR‐induced phosphorylations by prior MET inhibition. Following assay development, detectability of these assays was tested in representative phosphoproteome samples (data not shown). Of the 32 assays developed for sites selected from the screen, 20 endogenous peptides were detectable and selected for measurement. Second, by selecting known substrates in signaling networks downstream of MET, ATM, and the above‐mentioned kinases, we aimed to test the hypothesis that METi can modulate the DDR indirectly by modulating the activity of CDKs and RSKs. To visualize the final list of SRM assays used in the following experiments (and detailed in Table S4), we extracted from databases the known KSRs in these signaling networks and supplemented those with the predicted KSRs from our dataset (Fig. 2A).

Samples from the three cell lines mentioned above (Detroit 562, EBC‐1, and GTL‐16) were harvested following METi alone, IR alone, or a combination of treatments. By employing this diverse set of cell line models, we aimed to identify the signaling nodes and phosphorylations that are commonly regulated by MET signaling. Cells were collected at two time points (1 and 8 h post‐IR) to possibly distinguish between the early and the late DDR signaling elicited by IR alone or in combination with METi. Altogether, in 54 samples (three cell lines, six conditions, three independent replicates), 60 phosphopeptides were quantified with heavy reference standard peptides (Fig. 2B) and two phosphopeptides were quantified without a heavy reference. To assess the impact of the treatments, we analyzed the phosphorylation changes in the three cell lines, first comparing IR alone to untreated samples, and next comparing METi combined with IR to IR alone. Of note, MET Y1234&5 was detected in EBC‐1 and GTL‐16 and downregulated upon METi treatment, irrespective of IR, as expected (Fig. 2C), but below the limit of detectability in Detroit 562, unlike in the WB experiment (Fig. 1D).

The three cell lines responded similarly to IR (Fig. 2B), as measured by known IR‐induced phosphorylations such as NBN S343 (Fig. 2C), RAD50 S635, SMC1A S957, and TIF1B (also known as KAP1) S824. We also monitored several phosphorylations on PIKKs: While the detectability of ATM S1981 and ATR T1989 was at the limit, ATM S2996 was detectable and upregulated in all three cell lines upon IR. PRKDC S3205, an ATM‐dependent site (Neal *et al*., 2011), was also upregulated upon IR in all three cell lines, while PRKDC S2612 was not detectable. Of note, one phosphorylation selected from the discovery screen, CDN1B (also known as p27kip1) S140, was detected and upregulated by IR only in EBC‐1 cells, likely due to a high stable level of this protein (Shen *et al*., 2015).

While the response to IR was similar across all three cell lines, EBC‐1 and GTL‐16 responded differently to the combination of METi and IR (Fig. 2B). By using SRM on a larger sample set, we were able to resolve which phosphopeptides agreed with the patterns found in the discovery screen. Several phosphorylations that were upregulated by IR were further upregulated by prior METi treatment, in accordance with the quantitative pattern observed for cluster 2 in the discovery screen. Three particular phosphorylations were modulated in such a way in both cell lines but not in Detroit 562 cells: NUMA1 S395, TP53BP1 T855, and ACIN1 S243 (Fig. 2C and Table S4). Other IR‐induced phosphorylations were regulated by prior METi treatment only at specific time points or cell lines, including SMC3 S1083, PRKDC S3205 (both Fig. 2C), and RAD50 S635 (Table S4). While phosphopeptides mapping to SMC1A and NBN were ambiguously associated with both clusters 1 and 2 in the discovery data, the measured phosphorylation sites were regulated only by IR in the targeted experiment (Fig. 2B,C and Table S4). H2AX S139 (Fig. 2C), which was upregulated by IR in all three cell lines, was regulated by METi only in GTL‐16 at 8 h post‐IR. On the other hand, H2AX phosphorylated on both T136 and S139, a phosphopeptide selected from the discovery experiment, was upregulated by METi at both time points and in both EBC‐1 and GTL‐16 cells (Table S4). Last, the IR‐induced phosphorylation site CHEK1 S317 was downregulated by METi (Fig. 2C), in accordance with METi‐dependent decrease in CHEK1 S345 observed in the discovery experiment and the WB experiment (Fig. 1D). This downregulation most probably reflects our previous observation that prolonged METi downregulates CHEK1 protein levels in GTL‐16 and EBC‐1 cells (Mikami *et al*., 2015). On the other hand, protein levels of other DDR proteins remained unchanged (Fig. S3C). In sum, our SRM data confirmed that MET inhibition modulated specific IR‐induced phosphorylations.

### 3.3 Downregulation of CDK and RSK activities upon MET inhibition

As aforementioned, the discovery data suggested that MET inhibition leads to downregulation of CDKs and RSKs, but the data did not include enough phosphorylations to select from for SRM (Fig. S2). Therefore, to extend the discovery findings, and explore the relationship between these networks and IR, we included in our measurements selected surrogates of these kinases (Fig. 2A and Table S4). In GTL‐16 and EBC‐1 cell lines, we observed downregulation of CDK2 Y15 (Table S4), a phosphopeptide mapped also to CDK1 and CDK3, possibly due to downregulation of the CDK2 protein levels in EBC‐1, but not in GTL‐16 [Fig. S3C and previously reported (Zhang *et al*., 2014)]. Modulation of CDK activity was further attested by downregulation of STMN1 S25 (Fig. 2C) and S38 (Table S4) and P3C2A S259 (Table S4). RB1 S807 was detected only in EBC‐1 cells and downregulated by METi (Table S4). Interestingly, ATRIP S224, a CDK2 site important for ATR‐ATRIP activity (Myers *et al*., 2007), was also downregulated by METi only in EBC‐1 cells (Fig. 2C). Additionally, whereas CDN1B S10 was detected at a high level in EBC‐1 but was not regulated by METi, its low level observed in GTL‐16 was upregulated by METi, possibly due to protein stabilization (Table S4). Thus, EBC‐1 and GTL‐16, but not Detroit 562, may have evolved different signaling mechanisms employed for cell cycle arrest induced by METi.

The downregulation of phosphopeptides mapped to RPS6 and IF4B observed in the discovery data suggested downregulation of RSKs upon MET inhibition. This is likely due to the downregulation of one or numerous signaling networks governed by MET in these cell lines, such as the SRC‐PI3K‐AKT1‐mTOR, ERKs, JNKs, STAT. Extending on the downregulation of RPS6 phosphorylation analyzed by WB, we found by SRM that several signaling networks are modulated in EBC‐1 and GTL‐16, but not in Detroit 562 cells. In both EBC‐1 and GTL‐16 cells, numerous phosphorylations measured along the SRC‐PI3K‐AKT1‐mTOR network were downregulated by METi. From the discovery data, IF4B S422 was confirmed as downregulated by METi (Fig. 2C), along with phosphorylations on RPTOR, LARP1, GSK3, AKTS1, and AXA2L, as surrogates for mTOR, AKT, and SRC activities.

Importantly, none of these surrogate phosphorylations was modulated by IR in these cell lines. Yet, the inhibition of proliferation and metabolism networks, as observed here, can lead to indirect effects on the response to IR, by proteins that are regulated by proliferation but play a role in the DDR (and thus act as synergistic ‘platforms’). In EBC‐1 cells, based on SRM data, we could suggest several such phosphorylations—CHEK1 S280 (by RSKs), ATRIP S224 (by CDK2), and JUN S73 (by MAPKs). Such phosphorylations highlight the differences between EBC‐1 and GTL‐16 cells in response to METi, while other phosphorylations, such as NUMA1 S395, highlight their common response to prolonged METi followed by IR. The distinct lack of downstream response in Detroit 562 cells to METi further asserts the differences between these MET‐overexpressing cells.

### 3.4 Impact of METi on cellular DDR is largely ATM‐dependent and varies in time

**Fig. 3:**
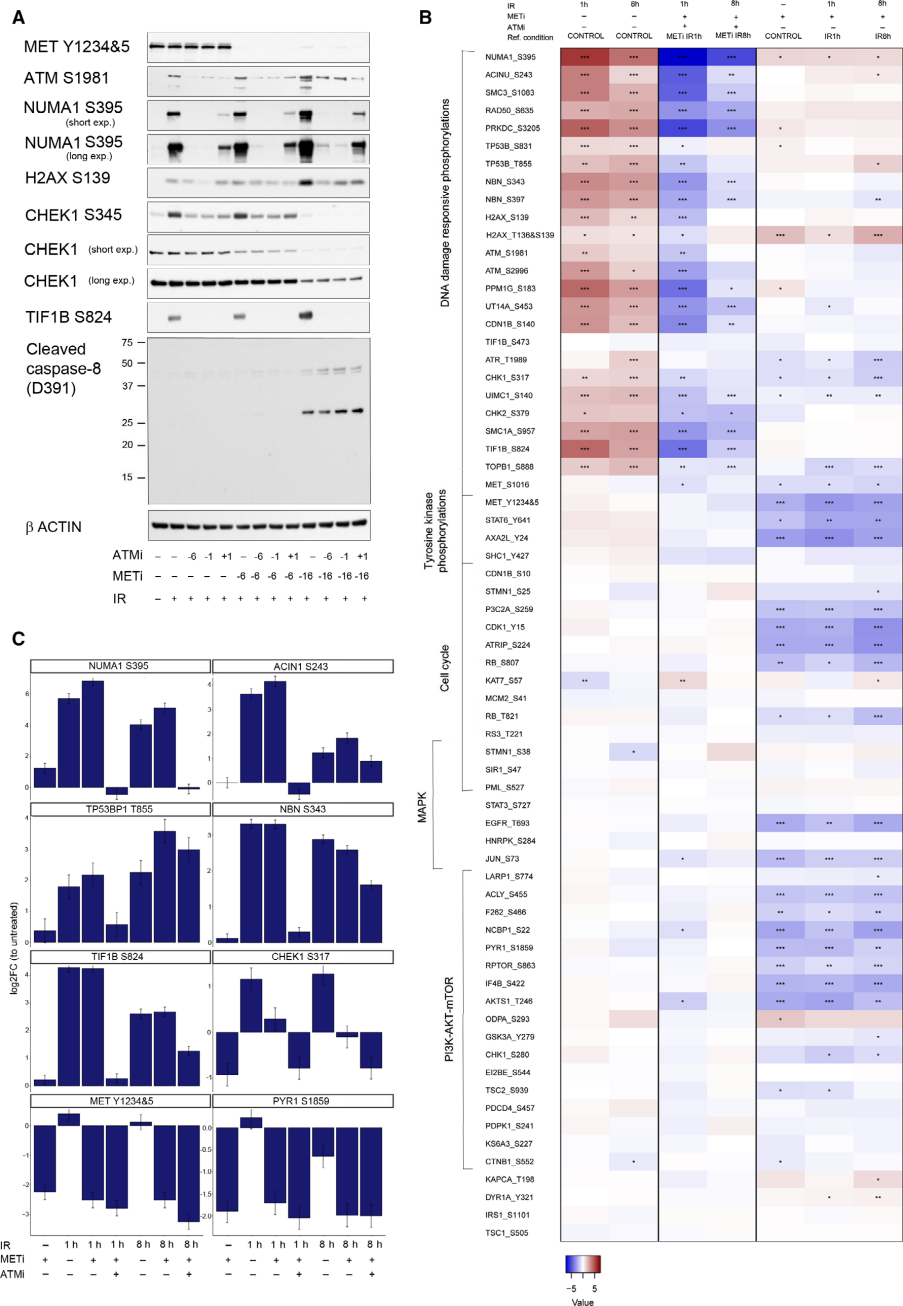
METi‐induced modulation of selected phosphorylations is time‐dependent and partially ATM‐dependent. (A) Impact of METi pretreatment duration (6 or 16 h prior to IR) and ATM activity (ATM was inhibited 6 or 1 h prior or 1 h post‐IR) on phosphorylation of MET, ATM, NUMA1, H2AX, CHEK1, and TIF1B, on cleavage of caspase‐8 and on CHEK1 protein levels. Cells were harvested 2 h post‐IR, and β‐actin was used as a loading control. (B) METi‐ and ATMi‐based modulation of cellular phosphorylations measured by SRM in EBC‐1 cells. Listed assays have been monitored in samples treated with ATMi, METi, IR, or the combinations (METi + IR; ATMi + METi + IR). Here are presented the results from statistical comparisons addressing three questions: Which are the significant changes in response to IR alone, compared with control (left); which are the significant changes in response to the combination METi + IR, compared with IR alone (right); and which are the significant changes in response to the combination ATMi + METi + IR, compared with METi + IR (middle). Log_2_FC (*n* = 3) is represented by a color code; adjusted *P*‐values by symbol: <0.0005, ‘***’, <0.005, ‘**’, <0.05, ‘*’. (C) Log_2_FC per condition is presented in comparison with control sample (untreated) for selected phosphorylation events in EBC‐1 cells upon IR (1 or 8 h) with or without METi and/or ATMi pretreatment as compared to untreated condition (*n* = 3).

Thus far, in the described experiments samples were all collected after a total 24 h of METi treatment (e.g., samples were collected 8 h after a 16‐h METi pretreatment). We aimed to further investigate the time dependency of the METi‐modulated phosphoproteome upon IR. In addition, based on the network analysis, as well as our previous data (Bensimon *et al*., 2010), we speculated that NUMA1 S395 would be an ATM‐dependent phosphorylation. To test these hypotheses, we treated EBC‐1 cells with combinations of METi, ATMi, and IR at several time points. While short METi pretreatments for 3 h (Fig. S4A) or 6 h (Fig. 3A) did not modulate NUMA1 S395 phosphorylation, we observed a considerable increase in phosphorylation when METi was added 16 h prior to IR (Fig. 3A). A similar pattern was found for H2AX S139 and TIF1B S824 (Fig. 3A and Fig. S3A). Phosphorylations of CHEK1 S345 and H2AX S139 were only partially ATM‐dependent (Fig. 3A). At the three time points post‐IR, NUMA1 S395 phosphorylation was considerably downregulated by ATMi administered prior or post‐IR, in a manner similar to TIF1B S824, a well‐characterized ATM substrate (Fig. S4B). NUMA1 S395 phosphorylation was dependent on the duration of MET inhibition and could also be induced by longer treatments with METi alone (Fig. S4C). Furthermore, at the 16 h pretreatment we also observed a decrease in CHEK1 protein levels, and, interestingly, also a cleavage of caspase‐8, both ATM‐independent (Fig. 3A). Of note, while proliferation was inhibited at earlier time points (Fig. S3B), apoptosis, as attested by cleaved caspase‐3, was observed only at later time points (Fig. S4D). Altogether, these results pointed to several signaling events related to DDR that covary, depend on the duration of MET inhibition, and are noticeable already at a 16 h pretreatment.

Of these events, we decided to focus on ATM and examine which other METi‐modulated phosphorylations are ATM‐dependent at this time point. EBC‐1 cells were pretreated with METi for 16 h and were harvested either 1 or 8 h post‐IR (for a total of 17 or 24 h, rather than 24 h in the previous SRM experiment). In addition to these conditions, cells that were pretreated with METi were treated also with ATMi for 1 h before IR to assess ATM dependency of the regulated phosphopeptides. In total, 67 phosphopeptides were quantified with heavy reference standard peptides in this second SRM dataset. Twelve phosphopeptides were added based on known substrates of ATM, CDKs, and RSKs, and five phosphopeptides were removed (see Table S4).

As in the first experiment, several phosphorylations were significantly upregulated by METi when combined with IR (Fig. 3B), including NUMA1 S395 (at all the assessed time points), ACIN1 S243, and TP53BP1 T855 (both at 8 h post‐IR; Fig. 3C). Moreover, all these phosphorylations were ATM‐dependent along with numerous known ATM substrates [such as NBN S343 (Fig. 3C) and SMC1A S957 (Fig. S4E)], supporting the hypothesis that these are ATM‐mediated phosphorylation sites. CHEK1 S317 phosphorylation was also METi‐ and ATMi‐ dependent (Fig. 3C), in an analogy to S345 assessed by WB (Fig. 3A). In addition, ATRIP S224 and ATR T1989 were both downregulated by METi (Fig. S4E), and S888 on TOPB1, an ATR interactor, was upregulated by IR and downregulated by both METi and ATMi (Fig. S4E). Doubly phosphorylated H2AX T136&S139 was upregulated by METi at 8 h but independent of IR or ATMi (Fig. S4E).

Beyond IR‐induced phosphorylations, the majority of METi‐dependent events that were described in the first experiment were also reproduced in this experiment. Downregulation of AKT‐mTOR‐RSK activities by METi was further strengthened by the additional assays measured in this experiment. In particular, we observed the downregulation of the RPS6KB1 substrate, PYR1 S1859 (Fig. 3C), also known as CAD. As discussed below, we hypothesize that this protein may be another example of indirect modulation of the response to IR following METi treatment.

### 3.5 Verification of METi modulation in a xenograft model

**Fig. 4:**
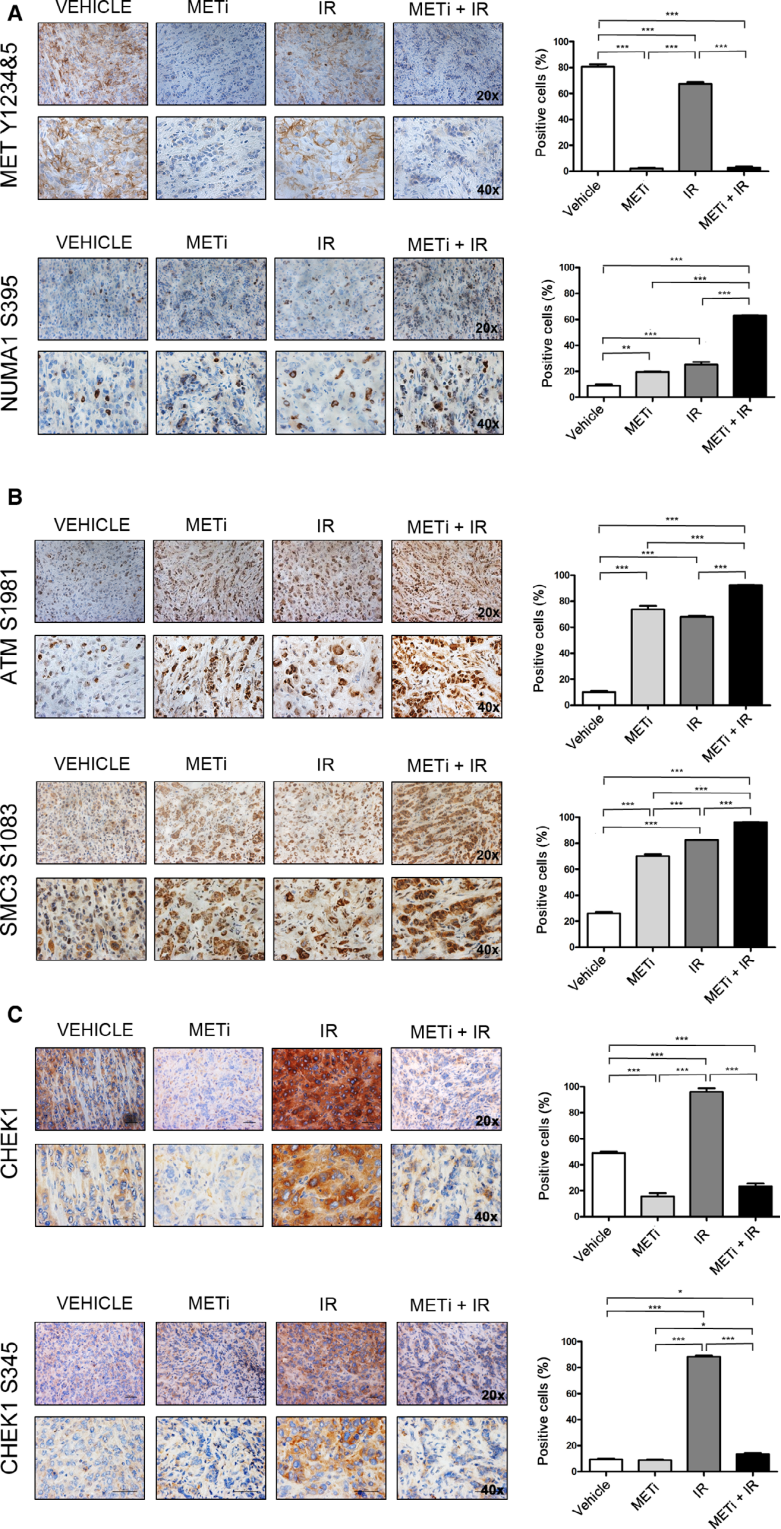
The relationship between METi and DNA damage‐related phosphorylations *in vivo*. IHC assessment in a xenograft tumor model of EBC‐1 cells. Animals were treated by oral administration of METi (15 mg·kg^−1^·day^−1^ for six consecutive days), local irradiation [a single dose (6 Gy) on day 3], or the combination of these two modalities. Following these treatments, the levels of mentioned proteins or phosphorylations sites were assessed in tumor tissues. Representative images (left panel) and quantification [% of positively stained area; mean (*n* = 3) ± SEM; right panel] of (A) MET Y1234&5, NUMA1 S395, (B) ATM S1981, SMC3 S1083, and (C) CHEK1 and CHEK1 S345 in EBC‐1 subcutaneous xenografts following IR, METi, or their combination. Statistical analysis was performed with the GraphPad Prism Software using one‐way ANOVA test (*P < 0.05; **P < 0.01; ***P < 0.001).

Overall, when examining how pretreatment with METi‐modulated IR‐induced phosphorylations, three patterns emerged in tissue culture: those unchanged, those upregulated by METi (as exemplified by NUMA1 S395), and those downregulated by METi (as exemplified by CHEK1 S345). Last, we aimed to examine *in vivo* the modulation by METi of these DNA damage‐related phosphorylations. We generated a xenograft tumor model by a subcutaneous injection of EBC‐1 cells into immunocompromised animals, and we treated them by oral administration of METi (15 mg·kg^−1^·day^−1^ for six consecutive days), local irradiation (a single dose of 6 Gy on day 3), or the combination of these two modalities. Following these treatments, the levels of MET Y1234&5, NUMA S395 (both Fig. 4A), ATM S1981, SMC3 S1083 (both Fig. 4B), and CHEK1 S345 (Fig. 4C) in tumor tissues were assessed by IHC. Surprisingly, we observed a significant increase in the number of ATM S1981‐positive and SMC3 S1083‐positive nuclei in tumor tissues originating from animals that received METi treatment alone as compared to untreated animals, and a further moderate increase in the combination treatment as compared to IR alone (Fig. 4B). In the xenografts from mice that were treated with both METi and IR, the levels of NUMA1 phosphorylation were significantly higher than in mice treated with one of the modalities alone (Fig. 4A). Consistently, CHEK1 protein levels and S345 phosphorylation were significantly reduced by IR combined with METi treatment as compared to IR alone (Fig. 4C). Taken together, particularly NUMA1 S395 and CHEK1 S345 were successfully able to distinguish between xenografts treated with IR versus those xenografts which were treated with the combination of METi and IR, in agreement with the results obtained by WB and SRM.

In summary, in the current study we have identified and validated phosphorylation changes associated with signaling networks at the intersection between MET and the DDR. Out of the multiple phosphorylations characterized by SRM, WB, and IHC, several candidates were demonstrated to be modulated by the sequential MET inhibition and IR. Regulation of two selected specific sites could be reproduced in *in vivo* settings and would be thus considered as potential markers for the MET‐DDR crosstalk in the context of future translational applications.

## 4 Discussion

Previous studies provided experimental evidence supporting a role for MET in the DDR (Bhardwaj *et al*., 2013; De Bacco *et al*., 2011; Medova *et al*., 2010; Medova *et al*., 2013a). In this report, we aimed to explore how MET inhibition modulates the cellular phosphorylation response to IR using a phosphoproteomic approach. In a discovery experiment, using immunoaffinity enrichment followed by MS, we identified hundreds of modulated phosphopeptides, on par with such experiments (Kirkpatrick *et al*., 2013; Matsuoka *et al*., 2007), including two METi‐modulated subsets with potential roles in DDR signaling: a subset of IR‐induced phosphorylations which are upregulated by prolonged prior METi treatment, and another subset of phosphorylations which are downregulated by METi regardless of IR. To explore these quantitative changes in multiple conditions, we selected several example candidates for further analysis using SRM. In addition, we selected from literature known substrates in these signaling networks, to generate new hypotheses regarding the relationships between MET inhibition and the DDR. In general, results obtained by SRM substantially expanded the results obtained by WB, and demonstrated good agreement with WB data, with few exceptions (e.g., TIF1B). It is important to note that in this study we focused solely on changes in protein phosphorylation and we cannot exclude the possibility that some of the observed changes are due to an underlying change in protein levels, exemplified in this study for CHEK1 and CDK2. Changes in protein abundance, or changes in both protein abundance and phosphorylation level, constitute interesting observations in their own right. Overall, as we discuss here, our approach has revealed multiple protein nodes involved in the DDR and modulated by prior inhibition of MET.

First, a few of the ATM‐dependent phosphorylations were upregulated by the prolonged MET inhibition combined with IR compared with IR alone, exemplified by NUMA1 S395 phosphorylation. NUMA1, such as ACIN1 and TP53BP1, is important in nuclear organization, and was shown to localize at several structures with key relevance to the DDR: NUMA1 localized with ATM at spindle poles during mitosis (Palazzo *et al*., 2014), at DNA damage sites to facilitate repair (Vidi *et al*., 2014), in the nucleolus to regulate rRNA transcription (Jayaraman *et al*., 2017), and at camptothecin‐stalled replication forks (Ribeyre *et al*., 2016). Furthermore, NUMA1 has also been demonstrated to have a role in apoptotic chromatin rearrangement in a caspase‐dependent manner (Dieker *et al*., 2012). While these proteins (e.g., NUMA1) have been extensively studied, very little is known about the ATM‐dependent phosphorylation events described here that attest to the complex roles these proteins play in DDR signaling. While the precise functional significance of these phosphorylation events remains to be established, these findings point to a novel aspect of the DDR: a particular class of putative ATM substrates, for which the degree of phosphorylation is not only ‘sensitive’ to the context of DNA damage (as ATM substrates are expected to be), but also ‘sensitive’ to the proliferative context in the perturbed cells.

Second, we demonstrated that phosphorylations on CHEK1, ATR, ATRIP, and TOPB1 were downregulated upon prolonged METi treatment combined with IR. The decrease in CHEK1 phosphorylation is likely due to a reduction in its protein levels reported previously (Mikami *et al*., 2015). Importantly, downregulation of CHEK1 has been demonstrated also in other models of oncogene addiction, such as EGFR, AXL, BCR‐ABL, JAK, and others (Balaji *et al*., 2017; Greve *et al*., 2015; Kurosu *et al*., 2013). Given the wealth of information about involvement of tyrosine kinases in regulation of the DDR (Mahajan and Mahajan, 2015), we anticipate that even more kinases are involved in CHEK1 regulation. Importantly, it would be expected that CHEK1 downregulation, and perhaps the modulation of the entire ATR pathway, would be modulated by METi‐dependent impairment of cell cycle checkpoints. Interestingly, although EBC‐1 is a p53‐deficient cell line and GTL‐16 cells express wt p53, they both display the same regulations of critical MET‐DDR phosphorylations reported here. Thus, while of utmost importance in DDR signaling (Fei and El‐Deiry, 2003), p53 status is not the main determinant of the reported phenomena.

Third, our discovery data suggested that METi leads to upregulation of a phosphopeptide on PRKDC containing the autophosphorylation site S2612, required for its activation (Ding *et al*., 2003). In agreement with our discovery data, Kirkpatrick *et al*. (2013) also found this phosphopeptide to be upregulated upon combined inhibition of MEK and PI3K, both inhibited by METi in our experiments. Despite our efforts, we were not able to detect this phosphorylation by WB or MS and cannot confirm or refute this observation. However, we did measure the well‐detectable site PRKDC S3205, which demonstrated modest but significant upregulation by the combination of METi and IR, suggesting a role of this kinase in the response to METi.

We noted that the changes in phosphorylations described here were more pronounced the longer the inhibition of MET lasts. This relationship would suggest that these are a consequence of prolonged processes that take place in cells, either as an indicator of an unresolved event (e.g., due to replication fork stalling) or as an early readout of an event to come (such as apoptosis). Accordingly, the combined treatment led to a rapid reduction in a proliferation marker, followed by an apoptotic response that was pronounced at later time points (Figs [Link], [Link]). One cellular process likely involved is the cell cycle progression—the longer the duration, the more likely it is that cells would have completed a (potentially improper) cellular division. We and others have already previously demonstrated that prolonged MET inhibition led to a G1 cell cycle arrest (Berthou *et al*., 2004; Medova *et al*., 2013b), and in accordance, we have measured several surrogates related to CDK activity, which are downregulated by METi, and may be indirectly involved in DDR regulation.

The discovery data have also led us to explore the role of RSKs governed by MET (in certain cell lines) which can also have roles at the intersection between cell cycle and DNA damage: RSK activity has been shown to modulate CHEK1 activity and the G2 cell cycle checkpoint by phosphorylation of CHEK1 on S280 (Grabocka *et al*., 2014; Ray‐David *et al*., 2013). We noted higher levels of CHEK1 S280 in GTL‐16 and EBC‐1 cells as compared to Detroit 562, although the molecular basis for this observation is unknown. Another interesting RSK phosphorylation site measured by SRM, which was downregulated by METi and might be at the intersection to DNA damage, is PYR1 S1859. This protein is an important enzyme in *de novo* pyrimidine synthesis from glutamine (Ben‐Sahra *et al*., 2013). While its observed downregulation might be a consequence of growth inhibition and cell cycle arrest, it could also represent a cause for DNA damage: Inhibition of this enzyme for > 12 h was shown to lead to DNA damage marked by phosphorylated H2AX (Hastak *et al*., 2008). It should be noted that PYR1 is an example of novel hypotheses generated by the SRM data, and we cannot claim in the scope of this study a causative role for METi‐dependent regulation of these proteins.

As another potential protein at the intersection between these signaling networks, we showed by WB that prolonged METi treatment led to caspase‐8 cleavage in EBC‐1 cells (Fig. 3A), which can either reflect a consequence of aforementioned events, or a mechanism in itself. In EBC‐1 cells, caspase‐8 cleavage was independent of the presence of DNA damage, unlike the caspase‐8 activation demonstrated previously to be induced by sequential inhibition of EGFR combined with doxorubicin in other cell lines (Lee *et al*., 2012). In line with a possible role for caspase‐8 in regulation of phosphorylation events, several of the sites that were modulated by METi were recently reported in a study that examined relationships between apoptotic activation of caspase‐8 and phosphorylations, and found that these are mediated at least in part by PRKDC (Dix *et al*., 2012). Taken together, proteins such as CHEK1, PYR1, and caspase 8 represent possible venues, presented in this study, by which MET inhibition can indirectly modulate the DDR.

Since all three cell lines employed in this study express considerably high levels of total MET protein, we reason that these phenomena are rather related to MET activity. As demonstrated in our experiments and by previous findings (e.g., Bertotti *et al*., 2009; Shen *et al*., 2015; Zhang *et al*., 2014), multiple signaling networks are governed by MET in both EBC‐1 and GTL‐16 cells, suggesting these as cell lines ‘addicted’ to the MET oncogene. ‘Oncogene addiction’ reflects a pharmacological proprietary (i.e., extreme sensitivity to oncogene inhibition; see Fig. 1B) of translational relevance that has been a topic of investigation and several models have been suggested that relate to the mechanisms of addiction at the molecular level (reviewed in Torti and Trusolino, 2011). We propose to reconcile the consequences of the prolonged processes in the context of DNA damage by referring to the ‘oncogene addiction’ phenomenon. In one such model, a strong oncogenic signal activates strong and fast proliferation signals (and their respective checkpoint signals). Meanwhile, the same oncogenic signal controls slow pro‐death signals. In the event of oncogene inhibition, the proliferation signals decay rapidly, exposing over time the activation of death signals. In this study, we identified and confirmed several signaling events at the intersection between MET addiction and the DDR. Of importance from a translational perspective, alterations in two such substrates can be clearly recapitulated in *in vivo* settings. Such prominent examples arising from these experiments could be employed in subsequent preclinical and early clinical studies evaluating MET targeting‐based radiosensitization protocols. In addition, given that signaling downstream of the MET proto‐oncogene is to a certain extent shared with other oncogenic RTKs, we anticipate that our findings could be eventually tested and potentially validated in tumors driven by the aberrant activation of these oncogenes as well.

Last, we believe that this study has demonstrated the benefit of targeted proteomics, most often applied not only in the context of biomarker verification, but also in phosphoproteomics (de Graaf *et al*., 2015; Kennedy *et al*., 2016). By testing multiple hypotheses from the discovery screen, combined with hypotheses selected from literature, we have expanded our understanding of MET signaling, to examine the cellular context in which these phosphorylation events take place. We hope that targeted proteomics, by any of the available measurement techniques, will facilitate the elucidation of complex cellular signaling networks, to enable the characterization of multiple candidates selected from large‐scale discovery experiments, translating those to confirmed hypotheses.

## 5 Conclusions

Experimental findings of earlier studies indicated a role for the MET receptor in the DDR. In this report, we explored two distinct METi‐modulated protein phosphorylation subsets with potential roles in DDR signaling: IR‐induced phosphorylations which are upregulated by prolonged prior METi treatment and phosphorylations which are downregulated by METi regardless of IR. Overall, we identified and confirmed several signaling events at the interface between MET and the DDR. Moreover, we have shown that prominent phosphorylations residing at this MET‐DDR interface, such as NUMA1 S395 or CHEK1 S345, occur also in *in vivo* settings and are thus of translational relevance.

Taken together, such proteins present possible venues by which MET inhibition can both directly and indirectly modulate the DDR, which would require further investigation. We suggest that these alterations are, at least in part, a consequence of prolonged processes that take place in cells, such as the impairment of cell cycle progression. We propose to reconcile the consequences of these prolonged processes in the context of DNA damage by referring to the ‘oncogene addiction’ phenomenon, and hypothesize that some of these findings may apply to other RTKs.

## Conflict of interest

A. Blaukat is listed as a co‐inventor on all patents related to Merck's c‐Met inhibitor listed in this manuscript. No potential conflicts of interest were disclosed by the other authors.

## Author contributions

ABe and MM designed the experiments, with input by YZ and RA. ABe, MM, JPK, PF, EO, AAG, SMR, and RR performed the experiments. ABe and MM wrote the manuscript, with contributions by RA, YZ, and DMA. RA, MM, DMA, YZ, and ABl provided resources.

## Supporting information


**Fig. S1.** Modulation of different phosphorylation motif‐containing substrates upon METi, IR or their combination at 10 min or 8 h post‐IR: (S/T)*QG (A), T*(D/E)X(D/E) (B), and T*XR (C). Arrows are pointing at some prominent phosphorylation changes of these substrates. MET autophosphorylation was blotted separately as a control for MET inhibition, and duplicated here from Fig. 1A. Enrichment of GO terms for all regulated proteins (D) and clusters 1 and 2 (E).

**Fig. S2.** Network of KSRs, in which edges represent KSRs predicted by networKIN (dashed), and KSRs known from PSP (solid). The network was restricted to the predicted kinases and their respective known kinases (where applicable), and organized by the clusters (Fig. 1B). The regulated phosphorylation sites are illustrated with the fold changes as bars and colored based on their cluster association.

**Fig. S3.** (A) Regulation of phosphorylation of MET and its downstream signaling molecules in EBC‐1 cells upon inhibiting MET, AKT (AZD5363, f.c. 10 µm), PI3K (GDC0941, f.c. 1 µm) or ERK (AZD6244, f.c. 10 µm) with or without IR (10 Gy, lysis 8 h post‐IR) was assessed by WB. β Actin was used as a loading control. (B) Histone H3 Ser10 phosphorylation following METi (16 h pretreatment prior to IR) alone and in combination with IR (10 Gy, lysis at post‐IR time points as indicated) in EBC‐1 and GTL‐16 cells. β Actin was used as a loading control. (C) Total protein levels of MET, 53BP1, CDK2, SMC3 and β Actin (used as a loading control) upon METi (24 h), IR (10 Gy, lysis 8 h post‐IR) and their combination (METi pretreatment 16 h prior IR, lysis 8 h post‐IR) in EBC‐1, GTL‐16 and Detroit 562 cells.

**Fig. S4**. Modulation of selected DDR‐related phosphorylations in EBC‐1 cells upon 3 h of pretreatment by METi prior IR (10 Gy) (A) and upon ATM inhibition (KU55933, 10 µm) prior or post‐IR in combination with METi (3 h pretreatment) (B). The cells were lysed 3 h post‐IR, β Actin was used as a loading control. (C) Time‐dependent phosphorylation of NUMA1 Ser395 following METi treatment (alone) in EBC‐1 cells. (D) Cleaved caspase‐3 levels following METi (16 h pretreatment prior to IR) alone and in combination with IR (10 Gy, lysis at post‐IR time points as indicated) in EBC‐1 and GTL‐16 cells. Staurosporine treatment (f.c. 1 µm for 17 h) was used as a positive control and β Actin was employed as a loading control. (E) Log_2_FC per condition are presented in comparison to control sample in EBC‐1 cells. Selected phosphorylation events were assessed in EBC‐1 cells upon IR (1 or 8 h) with or without METi and/or ATMi pretreatment as compared to untreated condition.

**Table S1.** MaxQuant and Perseus results for the two immunoaffinity experiments.

**Table S2.** PTMScan motifs final dataset used for analysis, including clustering results.

**Table S3.** Kinase prediction enrichment table.

**Table S4.** List of phosphopeptides measured by SRM with the statistical results.
