# Supplementary figures and images for: Deciphering MET‐dependent modulation of global cellular responses to DNA damage by quantitative phosphoproteomics
Source: Mol Oncol. 2020 May 13;14(6):1185–206. doi: 10.1002/1878-0261.12696 (PMC7266272; doi:10.1002/1878-0261.12696)

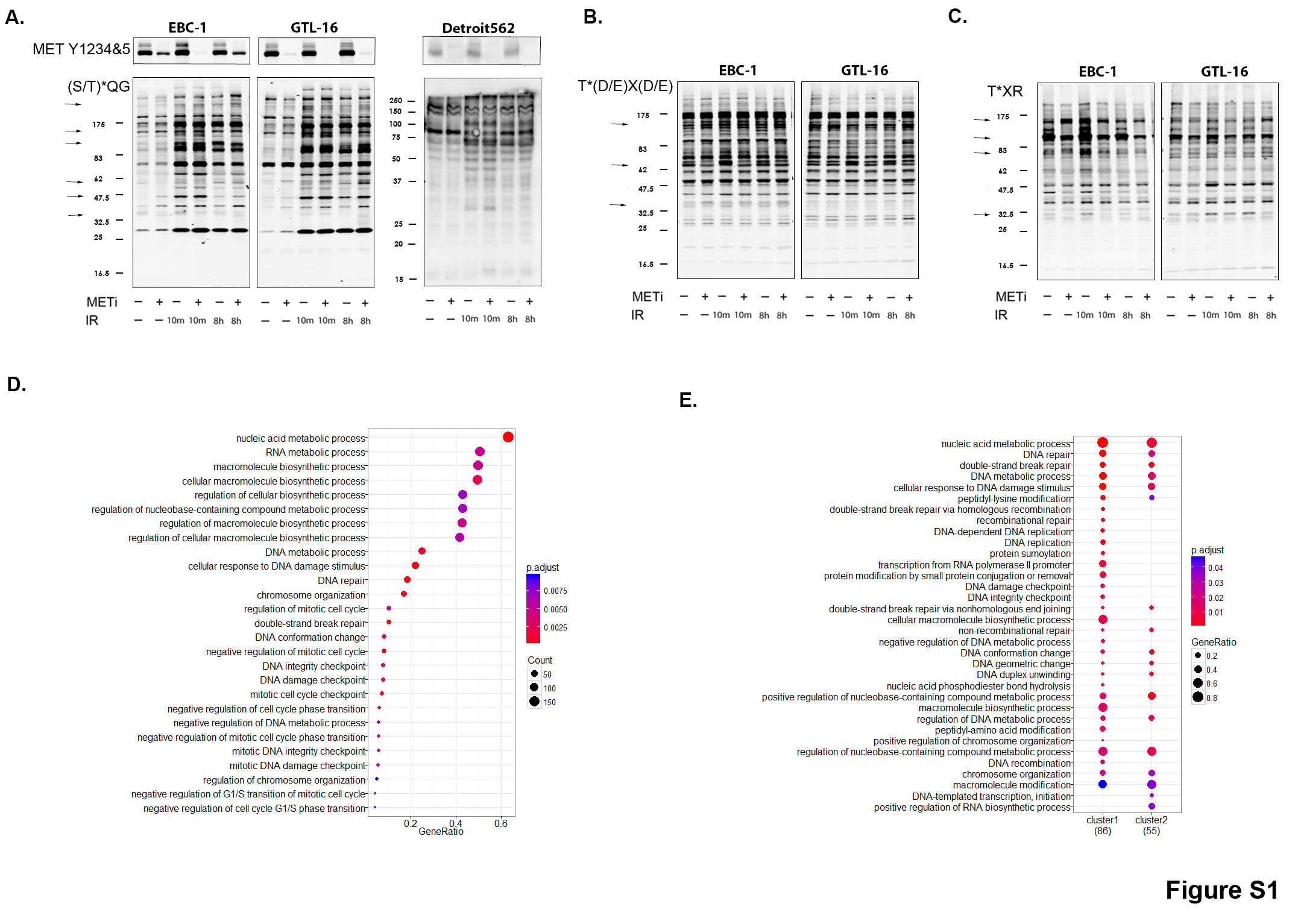

Supplement: Supplementary file 1 — Fig. S1. Modulation of different phosphorylation motif‐containing substrates upon METi, IR or their combination at 10 min or 8 h post‐IR: (S/T)*QG (A), T*(D/E)X(D/E) (B), and T*XR (C). Arrows are pointing at some prominent phosphorylation changes of these substrates. MET autophosphorylation was blotted separately as a control for MET inhibition, and duplicated here from Fig. 1A. Enrichment of GO terms for all regulated proteins (D) and clusters 1 and 2 (E). [file MOL2-14-1185-s001.tif]

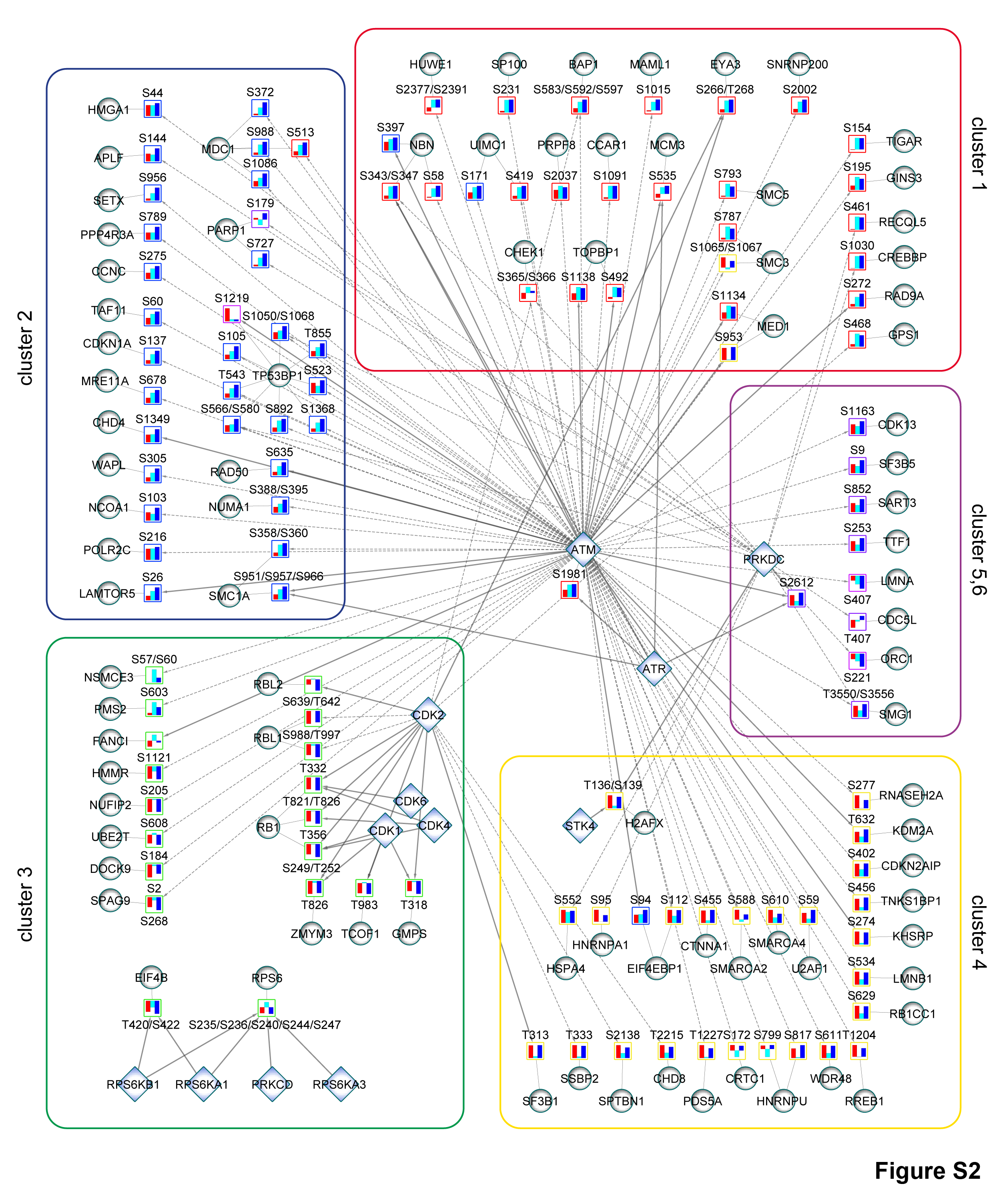

Supplement: Supplementary file 2 — Fig. S2. Network of KSRs, in which edges represent KSRs predicted by networKIN (dashed), and KSRs known from PSP (solid). The network was restricted to the predicted kinases and their respective known kinases (where applicable), and organized by the clusters (Fig. 1B). The regulated phosphorylation sites are illustrated with the fold changes as bars and colored based on their cluster association. [file MOL2-14-1185-s002.tif]

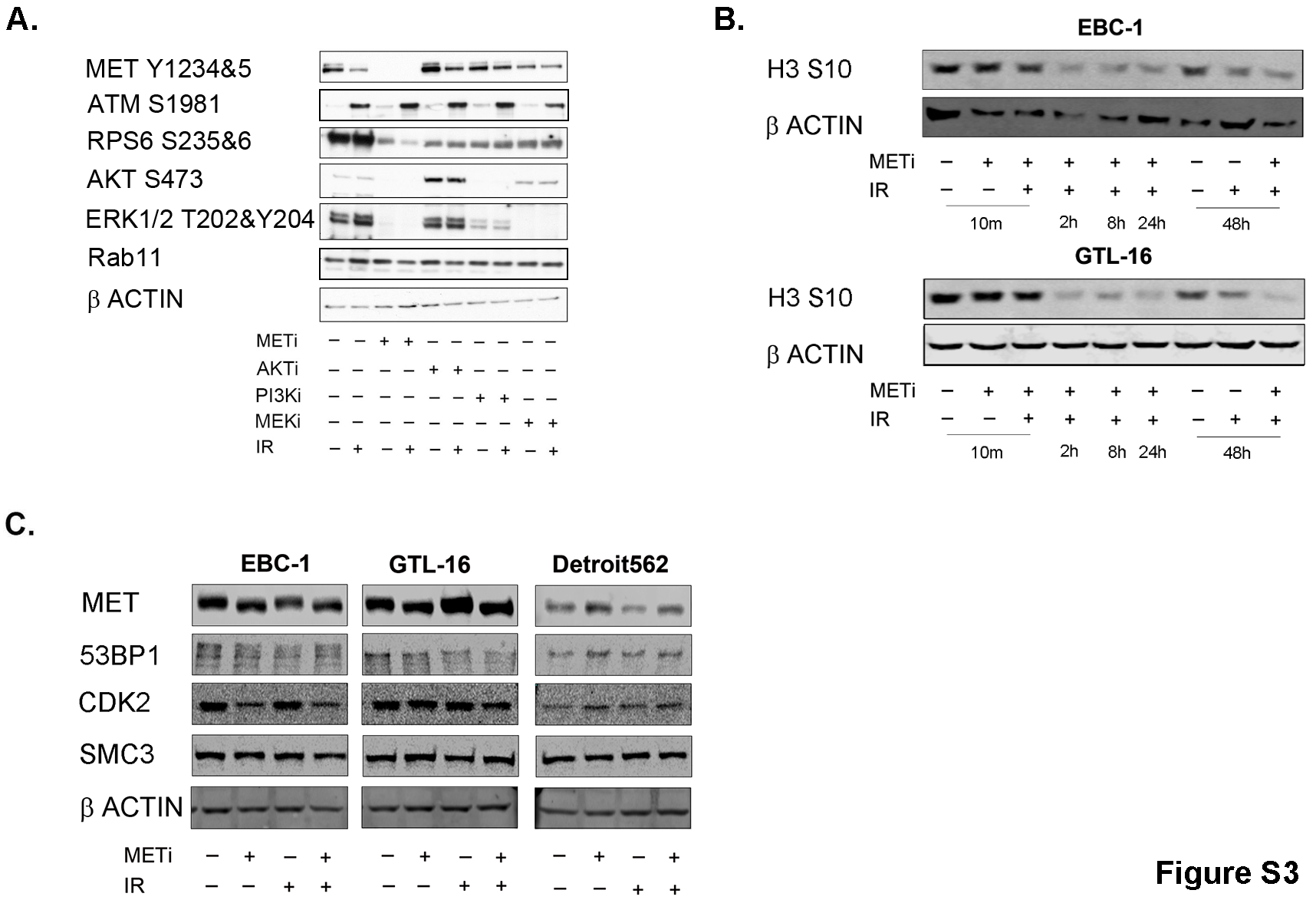

Supplement: Supplementary file 3 — Fig. S3. (A) Regulation of phosphorylation of MET and its downstream signaling molecules in EBC‐1 cells upon inhibiting MET, AKT (AZD5363, f.c. 10 µm), PI3K (GDC0941, f.c. 1 µm) or ERK (AZD6244, f.c. 10 µm) with or without IR (10 Gy, lysis 8 h post‐IR) was assessed by WB. β Actin was used as a loading control. (B) Histone H3 Ser10 phosphorylation following METi (16 h pretreatment prior to IR) alone and in combination with IR (10 Gy, lysis at post‐IR time points as indicated) in EBC‐1 and GTL‐16 cells. β Actin was used as a loading control. (C) Total protein levels of MET, 53BP1, CDK2, SMC3 and β Actin (used as a loading control) upon METi (24 h), IR (10 Gy, lysis 8 h post‐IR) and their combination (METi pretreatment 16 h prior IR, lysis 8 h post‐IR) in EBC‐1, GTL‐16 and Detroit 562 cells. [file MOL2-14-1185-s003.tif]

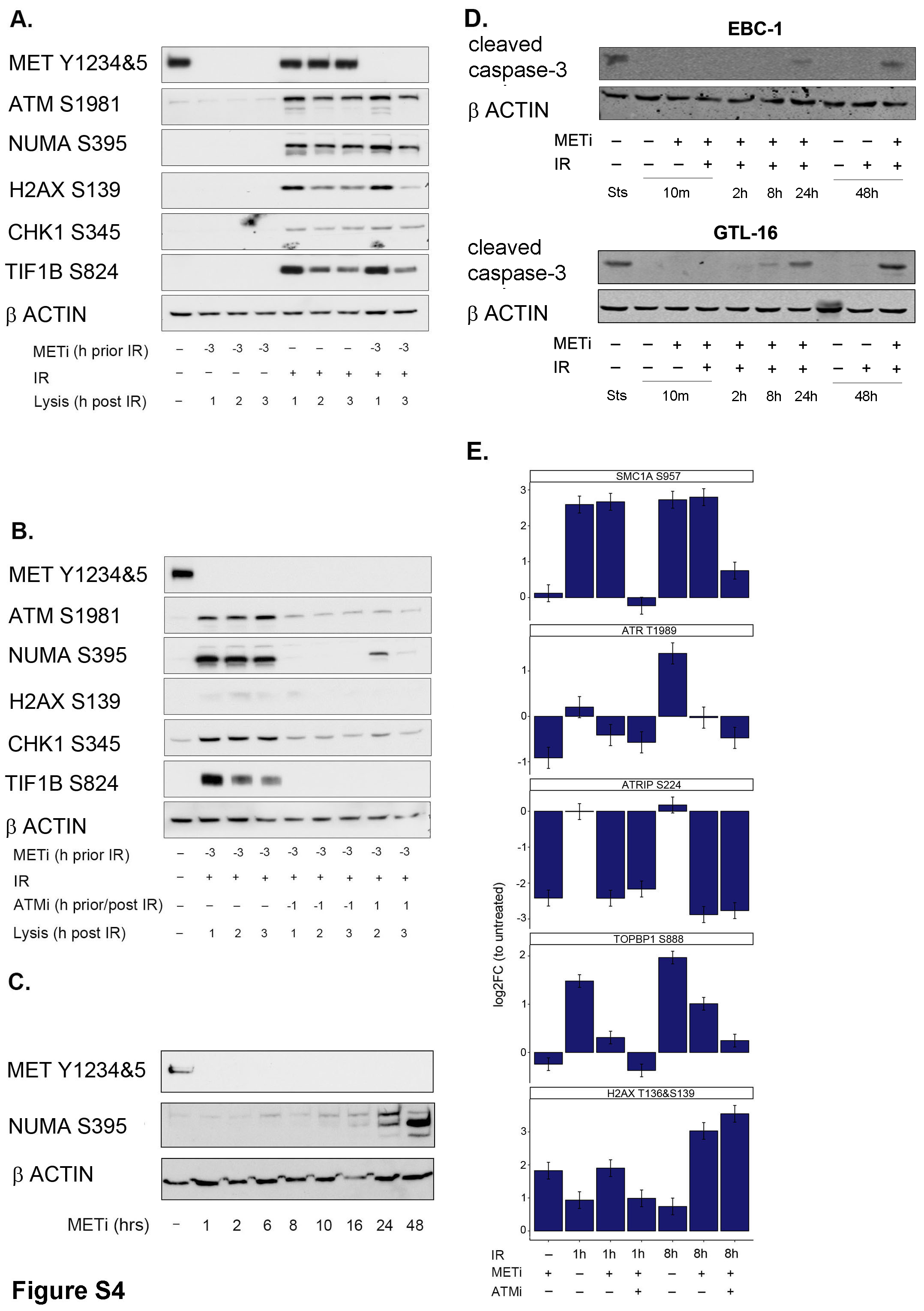

Supplement: Supplementary file 4 — Fig. S4. Modulation of selected DDR‐related phosphorylations in EBC‐1 cells upon 3 h of pretreatment by METi prior IR (10 Gy) (A) and upon ATM inhibition (KU55933, 10 µm) prior or post‐IR in combination with METi (3 h pretreatment) (B). The cells were lysed 3 h post‐IR, β Actin was used as a loading control. (C) Time‐dependent phosphorylation of NUMA1 Ser395 following METi treatment (alone) in EBC‐1 cells. (D) Cleaved caspase‐3 levels following METi (16 h pretreatment prior to IR) alone and in combination with IR (10 Gy, lysis at post‐IR time points as indicated) in EBC‐1 and GTL‐16 cells. Staurosporine treatment (f.c. 1 µm for 17 h) was used as a positive control and β Actin was employed as a loading control. (E) Log2FC per condition are presented in comparison to control sample in EBC‐1 cells. Selected phosphorylation events were assessed in EBC‐1 cells upon IR (1 or 8 h) with or without METi and/or ATMi pretreatment as compared to untreated condition. [file MOL2-14-1185-s004.tif]
